# Expanded potential stem cell media as a tool to study human developmental hematopoiesis in vitro

**DOI:** 10.1016/j.exphem.2019.07.003

**Published:** 2019-08

**Authors:** Adam C. Wilkinson, David J. Ryan, Iwo Kucinski, Wei Wang, Jian Yang, Sonia Nestorowa, Evangelia Diamanti, Jason Cheuk-Ho Tsang, Juexuan Wang, Lia S. Campos, Fengtang Yang, Beiyuan Fu, Nicola Wilson, Pentao Liu, Berthold Gottgens

**Affiliations:** a Department of Haematology, Wellcome & MRC Cambridge Stem Cell Institute, Cambridge, UK; b Wellcome Trust Sanger Institute, Hinxton, Cambridge, UK; c School of Biomedical Sciences, Li Ka Shing Faculty of Medicine, Stem Cell and Regenerative Medicine Consortium, University of Hong Kong, Hong Kong, China

## Abstract

Pluripotent stem cell (PSC) differentiation in vitro represents a powerful and tractable model to study mammalian development and an unlimited source of cells for regenerative medicine. Within hematology, in vitro PSC hematopoiesis affords novel insights into blood formation and represents an exciting potential approach to generate hematopoietic and immune cell types for transplantation and transfusion. Most studies to date have focused on in vitro hematopoiesis from mouse PSCs and human PSCs. However, differences in mouse and human PSC culture protocols have complicated the translation of discoveries between these systems. We recently developed a novel chemical media formulation, expanded potential stem cell medium (EPSCM), that maintains mouse PSCs in a unique cellular state and extraembryonic differentiation capacity. Herein, we describe how EPSCM can be directly used to stably maintain human PSCs. We further demonstrate that human PSCs maintained in EPSCM can spontaneously form embryoid bodies and undergo in vitro hematopoiesis using a simple differentiation protocol, similar to mouse PSC differentiation. EPSCM-maintained human PSCs generated at least two hematopoietic cell populations, which displayed distinct transcriptional profiles by RNA-sequencing (RNA-seq) analysis. EPSCM also supports gene targeting using homologous recombination, affording generation of an SPI1 (PU.1) reporter PSC line to study and track in vitro hematopoiesis. EPSCM therefore provides a useful tool not only to study pluripotency but also hematopoietic cell specification and developmental-lineage commitment.

## Highlights

• Expanded Potential Stem Cell Medium (EPSCM) stably maintains human pluripotent stem cells (PSCs).
• EPSCM-maintained human PSCs can undergo hematopoietic differentiation in vitro*.*
• A human SPI1-reporter PSC line enables study of in vitro hematopoiesis.

The in vitro derivation and stabilization of pluripotent stem cells (PSCs) has afforded unprecedented insights into early mammalian development 1, 2. With the ability to form all embryonic germ layers, PSCs have been particularly important for studying human development where direct investigation of embryogenesis is severely constrained. Within hematology, in vitro PSC hematopoiesis has provided a powerful model to study early specification events in hematopoietic cell formation as well as the different waves of developmental hematopoiesis that occur during embryogenesis 3, 4: the primitive wave, transient definitive wave, and definitive wave 5, 6. In vitro PSC differentiation has provided a tractable model to generate and study the various embryonic hematopoietic precursors [7], including investigation of the underlying molecular programs driving blood formation. A major aim behind these efforts has been to identify approaches to generate PSC-derived hematopoietic stem cells (HSCs) to provide an unlimited source for HSC transplantation, a curative therapy for a range of hematologic diseases 3, 8. In vitro PSC hematopoiesis is also being exploited to generate other hematologic cell types for blood transfusion and cellular immunotherapies 9, 10, as well as to model various hematologic diseases using patient-derived PSCs 11, 12, 13, 14, 15, 16.

One of the confounding issues in studying mammalian PSC differentiation is the difference in the culture conditions used for mouse and human PSCs. Mouse PSCs are leukemia inhibitory factor (LIF) dependent and are often cultured in “2iLIF” conditions (consisting of Mapk/Erk inhibitor PD0325901, GSK3 inhibitor CHIR99021, and LIF), which is thought to represent an in vitro equivalent of the embryonic blastocyst stage [17]. By contrast, most human PSC cultures are fibroblast growth factor (FGF) dependent (and LIF independent), approximately equivalent to the later epiblast stage (and mouse epiblast stem cells) [18]. Recently, several laboratories have described derivation of LIF-dependent human PSCs that represent a more naïve-like PSC state 19, 20, 21, 22, 23. However, to date most human PSC differentiation protocols start from FGF-dependent cultures 3, 24, 25, 26, whereas mouse PSC differentiation protocols start from LIF-dependent cultures.

We recently developed a novel chemical media formulation called Expanded Potential Stem Cell Medium (EPSCM) 27, 28, which combines inhibition of the MAPK, Src, and WNT/Hippo/TNKS1/2 signaling pathways, a glycogen synthase kinase 3-β inhibitor, and LIF. EPSCM maintained a relatively homogeneous population of mouse stem cells with expanded differentiation potential to both the embryonic and extra-embryonic lineages. Transcriptionally, these expanded potential stem cells (EPSCs), in addition to possessing a core pluripotency modulus, had features in common with the four- to eight-cell stage preimplantation embryo. Here, we report that the EPSC media (EPSCM) developed for mouse PSCs also supports human PSC maintenance and that EPSCM-maintained PSCs can differentiate into hematopoietic cell types.

## Materials and methods

### Human stem cell culture and reprogramming

Mouse EPSCs and human PSCs were cultured on mitomycin C-inactivated SNL feeder cells (SNL76/7) in EPSCM described previously 27, 28. Briefly, the media were composed of DMEM/F-12 medium (Invitrogen), high glucose, no glutamine, supplemented with 20% KnockOut serum replacement (KSR; Invitrogen), nonessential amino acids (MEM NEAA; Invitrogen), penicillin-streptomycin-glutamine (P/S/G; Invitrogen), 0.1 mM 2-mercaptoethanol (Sigma-Aldrich), 10^3^ U mL^–1^ human LIF (Merck Millipore), PD0325901 (Tocris, 1 μM), CHIR99021 (Tocris, 3 μM), JNK Inhibitor VIII (Tocris, 4 μM), SB203580 hydrochloride (Tocris, 10 μM), A-419259 trihydrochloride (Santa Cruz, 0.3μM), and XAV939 (Sigma-Aldrich, 5 μM). For routine bulk culture passaging in EPSCM, enzymatic dissociation with Accutase (Merck Millipore) was used. For human PSCs, addition of Y27632 dihydrochloride (Tocris, 10 μM) was necessary to improve survival during single cell passaging.

The import and use of human embryonic stem cells (hESCs) were approved by the Steering Committee for the UK Stem Cell Bank and by the Human Materials and Data Management Committee (HMDMC) of the Wellcome Trust Sanger Institute, Cambridge, UK. The hESC line, H1-ESC (WA01) [29], was cultured on a layer of mitotically inactivated mouse embryonic fibroblasts in hESC medium: DMEM/F-12 medium (Invitrogen), high glucose, no glutamine, supplemented with 20% KSR, MEM NEAA, P/S/G, 0.1 mM 2-mercaptoethanol (β-ME; Sigma-Aldrich), and 10 ng mL^–1^ FGF2 (Invitrogen). To convert hESCs to EPSCM, individual hESCs were seeded on SNL76/7 feeder cells in EPSCM at a low cell density (1 × 10^2^ cells cm^–2^).

For *piggyBac* (PB) transposon-based six-factor reprogramming of human dermal fibroblast cells (HDF) [30], transfection was performed using an Amaxa machine (program U-020). The DNA mixture for transfection of HDFs comprised 2.0 μg of PB-TRE-hOCKS, 1.0 μg PB-TRE-RL, 1.0 μg PB-EF1a-transposase, and 1.0 μg PB-EF1a-rtTA. The hOCKS and RL were made with human complementary DNAs of OCT4, cMYC, KLF4, SOX2, RARG, and LRH1 linked by 2A peptides. After electroporation, HDFs cells were seeded in M15 media: KnockOut DMEM (Invitrogen), high glucose, no glutamine, supplemented with 15% fetal bovine serum (FBS; Hyclone), MEM NEAA, P/S/G, 0.1 mM β-ME (Sigma-Aldrich), and 10^3^ U mL^–1^ human LIF (Merck Millipore), supplemented with ascorbic acid (Sigma, 50 μg mL^–1^) on mitomycin C-inactivated SNL76/7 feeder plates for 24 hours. From the second day, transfected cells were cultured in M15 media supplemented with vitamin C (50 μg/mL, Sigma) and doxycycline (1.0 μg/mL, Clontech). When colonies emerged 10-15 days after electroporation, the media was switched to EPSCM for a further 6-8 days before picking.

For neural stem cell (NSC) episomal (integration-free) six-factor reprogramming [30], BRC1019, a human fetal neural stem cell line (a gift from Dr. Colin Watts), was transfected using an Amaxa machine (Lonza) (program A-033). The DNA mixture for transfection of NSCs comprised 9.0 μg pCEP-EF1a-hOCK and 6.0 μg pCEP-EF1a-hRL, or 6.0 μg pCEP-EF1a-hOCK alone was used for successful reprogramming because NSCs already express high levels of endogenous SOX2. After electroporation, NSCs were seeded in M15 media supplemented with vitamin C (50 μg/mL, Sigma) on mitomycin C–inactivated SNL76/7 feeder plates. The primary iPSC colonies were then processed as above.

The human EPSCM PSC lines used in this study were EC2-EPSCM (XX) and EC5-EPSCM (XX) (transgene independent iPSCs derived from fetal NSCs using episomal six-factor reprogramming), PB-EPSCM (XY) (iPSC derived from adult HDFs using *piggyBac* transposon mediated transposition of the six-factor reprogramming constructs), and H1-EPSCM (XY) (converted H1-ESCs).

### In vitro human hematopoietic differentiation

Human PSCs maintained in EPSCM were differentiated when 50%–90% confluent and at least three passages after thawing. ESPCM was refreshed 3 hours before dissociation. Human PSCs were washed once with PBS and dissociated using Accutase for 5–10 minutes at 37°C. The dissociation was quenched by addition of 10 × the Accutase volume of basal media (BM): DMEM/F12, 20% KSR, MEM NEAA, P/S/G, 0.1 mM β-ME, and 10 μM Y27632. A single cell suspension was generated by gentle pipetting. To deplete SNL feeder cells, the cell suspension was transferred back onto plates/dishes and incubated at 37°C for 45 minutes. Unattached PSCs were then gently removed from the plate and pelleted at 300 *g* for 3 minutes. PSCs were resuspended at a concentration of 1 × 10^5^ cells/ml and 3 mL plated into Ultra-Low Attachment 6-well plates (Corning) and allowed to form embryo bodies (EBs) at 37°C with 5% CO_2_ for 48 hours. EBs were collected into 50 mL tubes and allowed to settle by gravity before BM was removed. EBs were resuspended in the same volume of human differentiation media: KnockOut-DMEM supplemented with 20% FBS (Hyclone; batched tested for human EPSC differentiation), P/S/G, 0.1 mM β-ME, 0.3 mg/mL human transferrin, and 0.3 mM ascorbic acid (based on media composition described previously [31]). EBs were replated back in the same Ultra-Low Attachment 6-well plates and incubated at 37°C with 5% CO_2_, and media replaced every 3–4 days. EBs up to 8 days in culture were dissociated using TrypLE only, whereas EBs more than 8 days in culture were dissociated using collagenase type I (Stem Cell Technologies) followed by TypLE to generate a single cell suspension. Colony forming assays were performed using H4435 Methocult (Stem Cell Technologies) according to manufacturer's instructions. Colony forming units (CFUs) were counted based on visual identification of CFU-M, CFU-G, CFU-GM, CFU-Mix, and BFUe.

### Flow cytometry analysis

Cells to be stained with antibodies were first Fc-blocked using purified anti-CD32/16 antibody (Biolegend), then stained with fluorophore-labeled antibodies (Supplementary Table E1, online only, available at www.exphem.org) in fluorescence-activated cell sorting (FACS) buffer (PBS supplemented with 2% heat-inactivated FBS) for 30 minutes at 4°C. Samples were washed twice with FACS buffer and resuspended in 500 μL FACS buffer supplemented with 0.5–1 μg/ml 4′,6-diamidino-2-phenylindole (DAPI; used as a viability stain) and analyzed on an LSRFortessa cell analyzer (Becton Dickinson) using single antibody stains to compensate. Flow cytometry results were analyzed using FlowJo software.

**Table S1:** Flow Cytometry Antibodies

**Anti-human antibodies**		
***Antibody***	***Clone***	***Company***
Fc block	TruStain FcX	Biolegend
CD34-PE	581	Biolegend
CD34-BV605	581	Biolegend
CD34-BV510	581	Biolegend
CD45-AF647	HI30	Biolegend
CD43-PE	1G10	BD
CD43-APC-H7	1G10	BD
CD41-PE-Cy7	HIP8	Biolegend
CD235a-FITC	HIR2	Biolegend
FLK/KDR-AF647	7D4-6	Biolegend
CD140a-PE	Alpha-R1	BD

**Anti-mouse antibodies**		
***Antibody***	***Clone***	***Company***
CD16/32 (Fc block)	93	BD
CD41-PE-Cy7	MWReg30	Biolegend
CD45-APC	30-F11	Biolegend
Ter119-FITC	TER119	BD

List of flow antibodies used for flow cytometry in this study.

### Gene targeting

Targeting vectors used to generate the ROSA26-SA-H2B-Venus-PGK-Puro and SPI1-2A-H2B Venus-EF1α-Puro reporter lines were all made using *Escherichia coli* recombineering, as described previously [32]. SA-H2B-Venus-PGK-Puro and 2A-H2B-Venus-EF1α-Puro constructs were gifts from Dr. Manousos Koutsourakis and Dr. Bill Skarnes, Sanger Institute. For targeting, PSCs were washed with PBS and dissociated using Accutase. After dissociation, cells were collected, counted, and resuspended in EmbryoMax ES Cell Electroporation Buffer (Merck Millipore). For one electroporation, 5 μg Cas9 expression vector (George Church Lab, Addgene), 5 μg guide RNA expression vector (George Church Lab, Addgene) and 10 μg of targeting vector were mixed with 1 × 10^7^ cells and electroporated with Biorad Gene Pulser using a condition of 320 V, 250 μF. After electroporation, the cells were plated onto SNL76/7 feeder plates in EPSCM supplemented 10 μM Y27632 for 24 hours. Puromycin (1.0 μg/mL) selection was performed 48 hours after electroporation. When drug-resistant colonies emerged, the medium was switched to EPSCM for an additional 2 days before picking.

### In vitro mouse hematopoietic differentiation

ESCs were differentiated using the EB formation method, as described previously 33, 34. Colony forming assays were performed using M3434 Methocult (Stem Cell Technologies) according to manufacturer's instructions.

### Immunostaining and imaging

Cells were fixed in 4% paraformaldehyde/PBS solution, blocked in PBS solution with 3% serum, 1% bovine serum albumin, and 0.1% Triton, and incubated with primary antibodies at 4°C overnight. Cells were rinsed and incubated with Alexa 488 or Alexa 594 conjugated secondary antibodies for 1 hour in the dark at room temperature. Cells were counterstained with DAPI. Antibodies used in this study are listed in Supplementary Table E2 (online only, available at www.exphem.org). Immunofluorescence stained samples were examined with a Leica DM5000B microscope equipped with narrow bandpass filters for Cy3.5, FITC, and DAPI fluorescence. Images were captured via a monochrome digital camera (ORCA-03G, Hamamatsu) and processed with the SmartCapture software (Digital Scientific UK). Alternatively, samples were examined with an Olympus IX81 microscope with narrow bandpass filters for FITC, Cy3.5, and DAPI fluorescence. Images were captured with a monochrome digital camera and processed with Cell^D software.

**Table S2:** Immunofluorescence Antibodies

Category	Antibody	Company	Catalog Number	Dilution
Primary Antibody	OCT4	Abcam	Ab1985	1:250
	OCT4 (C10)	Santa-Cruz	SC-5279	1:100
	SOX2	R & D Systems	MAB2018	1:100
	NANOG	Abcam	Ab80892	1:100
	SSEA3	Gift from Professor Peter Andrews, University of Sheffield. Use at 1:10		
	SSEA4			
	TRA-181			
Secondary Antibody	Alexa Fluor 488 and Alexa Fluor 594 (for IF)	Invitrogen		1:500 - 1:1000

List of antibodies used for immunofluorescence in this study.

### Cell cycle analysis

Click-iT Plus EdU Alexa Fluor 647 Flow Cytometry Assay Kit (Life Technologies, C10634) was used for cell cycle analysis according to manufacturer's instructions. EdU incorporation (Alexa Fluor 647 labeled anti-EdU antibodies) was measured with DNA content (DAPI) in fixed and permeabilized cells.

### Quantitative reverse transcription polymerase chain reaction

Total RNA was isolated using the RNeasy Mini Kit (Qiagen) for cultured cells. RNA was subsequently quantified and treated with gDNA WipeOut to remove genomic DNA. Complementary DNA was prepared using the QuantiTect Reverse Transcription Kit (Qiagen). TaqMan Gene Expression Assays (Life Technologies) (Supplementary Table E3, online only, available at www.exphem.org) and ABsolute Blue qPCR ROX Mix (ABgene) were used for probe-based quantitative polymerase chain reaction (qPCR) assays. All qPCR reactions were performed on the ABI 7900 HT Sequence Detection System (Life Technologies). Gene expression was determined relative to GAPDH using the ΔCt method. Data are shown as the mean and standard deviation.

**Table S3:** qPCR primer/probe sets

Gene Name	Applied Biosystems Catalog Number
CDX2	Hs01078080_m1
FGF4	Hs00173564_m1
GAPDH	4326317E
GATA6	Hs00232018_m1
GSC	Hs00418279_m1
NANOG	Hs02387400_g1
OCT4	Hs00999634_gH
PAX6	Hs01088114_m1
PRDM14	Hs01119056_m1
SOX1	Hs01057642_s1
SOX17	Hs00751752_s1
SOX2	Hs00602736_s1
STELLA	Hs01931905_g1
T	Hs00610078_m1

List of qPCR primer/probe sets use for qPCR analysis in this study.

### In vivo pluripotency assay by teratoma formation

All mouse experiments were performed in accordance with the UK's 1986 Animals and Scientific Procedures Act and local institute ethics committee regulations. NSG mice were subcutaneously injected with 5 × 10^6^ PSCs in 100 μL PBS containing 30% Matrigel in the dorsal flank (NOD.Cg-Prkdcscid Il2rgtm1Wjl/SzJ, The Jackson Laboratory). Teratomas developed within 4–8 weeks. The mice were culled using schedule 1 methods once the teratoma reached the legal limit (1.2 mm^2^) as per Home Office guidelines. Teratomas were dissected and fixed in 10% buffered formalin phosphate for at least 24 hours before paraffin embedding, sectioning, and hematoxylin-eosin staining. All slides were evaluated by a histopathologist.

### RNA-seq analysis

RNA-seq was performed using the SmartSeq2 protocol [35], with an in-house pipeline described previously [36]. Fifty cells were directly sorted into lysis buffer using an Influx Cell Sorter (Becton Dickenson), and next-generation sequencing using a HiSeq4000 at a depth of ∼4.5 million reads/sample (with ~2.3 million reads mapped to exons). Reads were aligned to human genome reference (hg19) using GSNAP (parameters: –n 1 –Q –N 1) and reads overlapping exons (ENSEMBL release 81) were counted using HTSeq. Samples passed quality control based on number of mapped reads, mappability, fraction of mitochondrial reads, and number of expressed genes. Differential expression was performed using the DESeq2 package [37] using <0.05 false discovery rate (FDR) and >1 log_2_(fold change) thresholds for pairwise comparisons. To detect unique marker genes for each condition in Figure 3F, we performed differential expression of each group against the remaining samples combined using <0.1 FDR and > 0.5 log_2_(fold change) thresholds. Top 15 upregulated genes, based on adaptive t prior shrinkage [38] of log_2_(fold change), are shown. Gene set enrichment analysis was performed using EnrichR [39]. Data sets are available on GEO (GSE130662).

## Results

**Figure E1:**
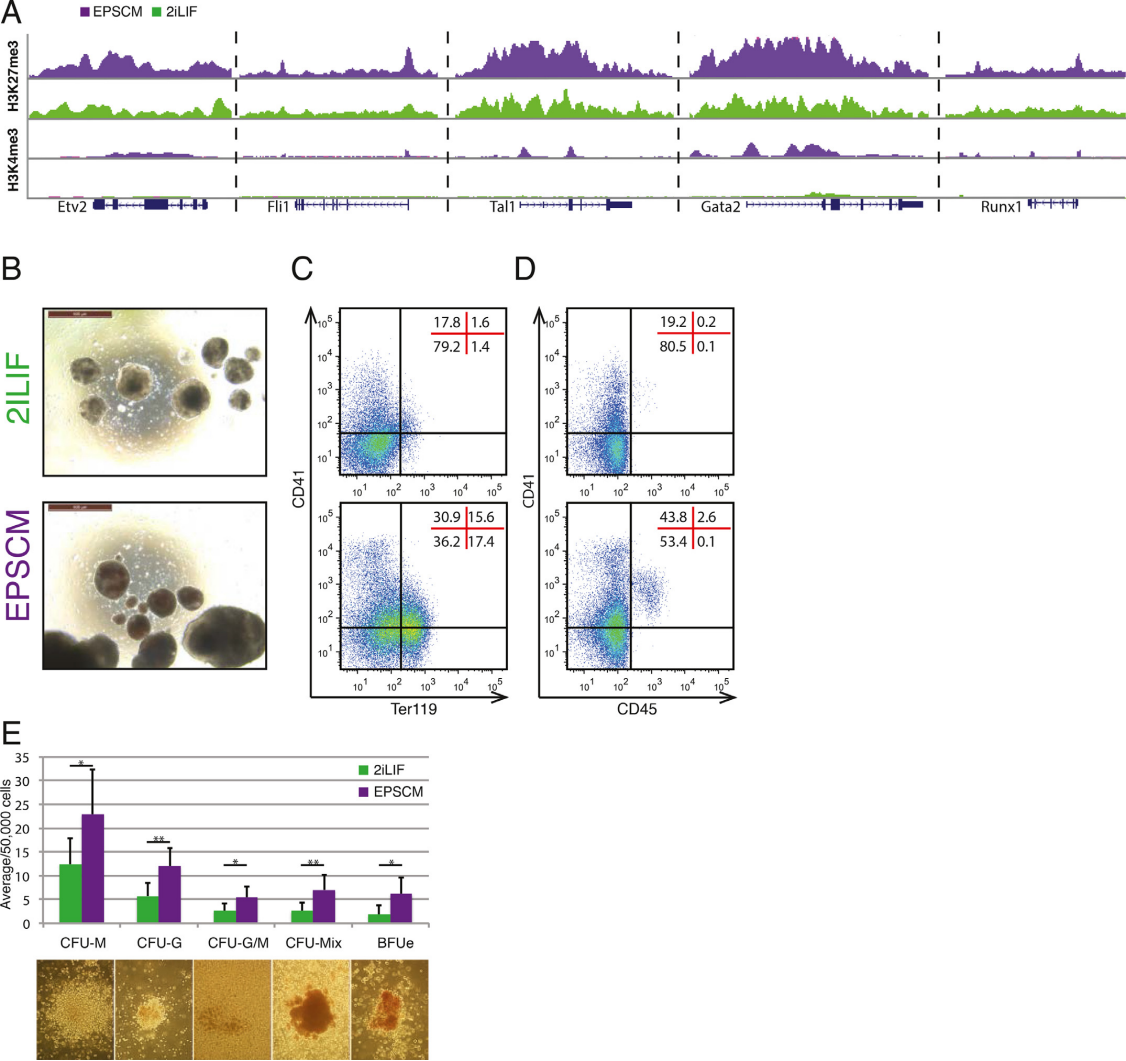
Mouse EPSCs efficiently undergo developmental hematopoiesis in vitro, related to Figure 1. (**A**) UCSC Genome Brower [57] screenshots of H3K27me3 (top) and H3K4me3 (bottom) ChIP-seq in DR10-EPSCM (in purple) and 2iLIF-DR10 PSCs published previously [40], in the following gene loci (from left to right): *Etv2, Fli1, Tal1, Gata2,* and *Runx1*. (**B**) Representative images of day 8 EBs derived from DR10 ESCs maintained in 2iLIF (top) or EPSCM (bottom). (**C** and **D**) Representative flow cytometry plots of day 8 EB cells from 2iLIF-DR10 (top) and EPSCM-DR10 PSCs (bottom), showing (**C**) CD41 versus Ter119 and (**D**) CD41 versus CD45. Distribution of cells within quadrant/gates is shown as percentages. (**E**) Average numbers of hematopoietic colonies from 5 × 10^4^ day 8 EB cells differentiated from 2iLIF-DR10 (in green) and EPSCM-DR10 (in purple) PSCs formed in methylcellulose supplemented with stem cell factor, interleukin-3 (IL-3), IL-6, and erythropoietin. Data are mean ± standard deviation from seven assays from three biological replicates. Statistically significant changes (Student's *t* test) in colony number denoted by * *p* < 0.05 and ** *p* < 0.01. Images of representative hematopoietic colonies scored are displayed.

One of the notable differences between mouse PSCs cultured in 2iLIF and EPSCM was a significant increase in the number of bivalent chromatin domains in EPSCM [27] (6224 vs. 3968). We noticed that this included several hematopoietic transcription factor gene loci [40], including *Etv2, Fli1, Tal1, Gata2*, and *Runx1* (Supplementary Figure E1A, online only, available at www.exphem.org). Based on this molecular signature, we hypothesized that EPSCM-cultured PSCs may readily undergo in vitro hematopoiesis. Consistent with this idea, 8-day EB differentiation of mouse DR10 PSCs displayed greater hematopoietic cell commitment when initiated from EPSCM than from 2iLIF culture conditions (Supplementary Figure E1B, online only, available at www.exphem.org). EPSCM-derived EBs contained a higher proportion of blood cells than 2iLIF-derived EBs, including Ter119^+^ erythrocytes (∼30% vs. ∼3%; Supplementary Figure E1C, online only, available at www.exphem.org) and CD41^+^CD45^+^ mature definitive hematopoietic cells (∼2.5% vs. 0.2%; Supplementary Figure E1D, online only, available at www.exphem.org). Methylcellulose CFU assays further confirmed EPSCM-derived EBs contained more hematopoietic progenitor cells (HPCs), generating approximately twice as many CFUs (Supplementary Figure E1E, online only, available at www.exphem.org). Given the efficiency of this system to generate hematopoietic cells from mouse EPSCs, we wondered whether the same approaches could be used to develop a simple system to study human developmental hematopoiesis in vitro.

**Figure 1:**
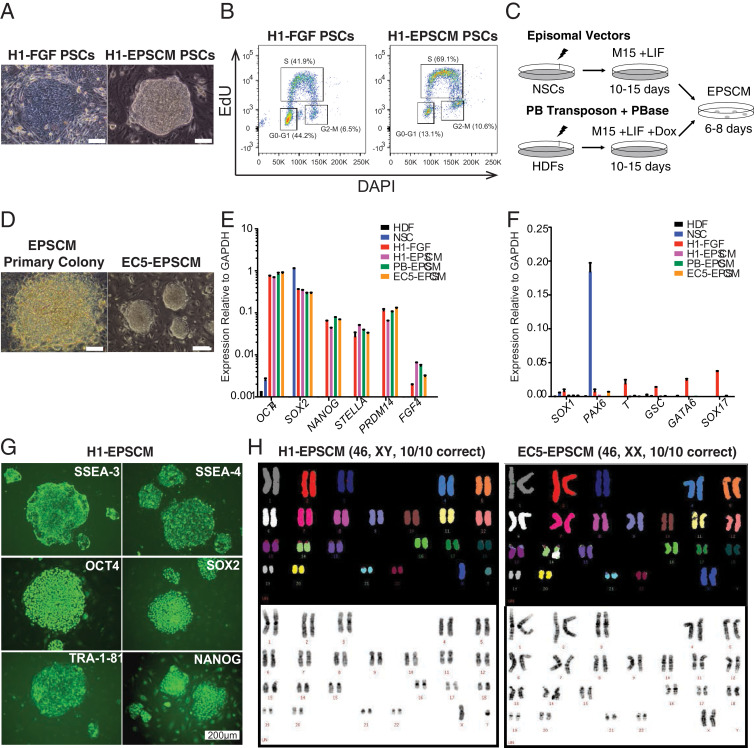
Generation of human PSCs in EPSCM**. (A)** Representative images of H1-FGF (left panel) or H1-EPSCM (right panel) PSC colonies (scale bar = 50 μm). **(B)** Cell cycle analysis of H1-FGF and H1-EPSCM (passage 15 in EPSCM) PSCs. Data representative of three biological replicates. **(C)** Graphical representation of the reprogramming strategy using episomal or *piggyBac* transposon vectors for PSC reprogramming from neural stem cells (NSCs) and human dermal fibroblasts (HDFs), respectively. Primary colonies appeared after 10–15 days, at which time the media was switched to EPSCM for an additional 6–8 days before picking. **(D)** Images of a primary PSC colony in EPSCM (left panel) and established EC5-EPSCM PSCs (right panel) (scale bar = 100 μm). **(E, F)** Expression of pluripotency and lineage markers genes in H1-FGF, H1-EPSCM, EC5-EPSCM, and PB-EPSCM PSCs. Parental HDFs and NSCs were used as control. Relative expression of these genes was normalized to *GAPDH*. Data are mean ± standard deviation. Data are representative of three biological replicates. **(G)** Immunostaining of H1-EPSCM PSCs for the cell surface markers SSEA3, SSEA4, and Tra-181 and the pluripotency transcription factors OCT4, SOX2 and NANOG (scale bar = 200 μm). **(H**) Spectral karyotype of H1-EPSCM and EC5-EPSCM PSCs at passage 12 and passage 15, respectively.

To test this hypothesis, we first needed to establish stable cultures for human ESCs/iPSCs in EPSCM. Because mouse PSCs could be converted from 2iLIF to EPSCM within several passages and because many of the signaling pathways that are active in preimplantation embryos are conserved between mouse and human, we initially attempted to convert the FGF-cultured hESC line WA01 H1 29, 41 (here termed H1-FGF). Dissociated H1-FGF were seeded in EPSCM on SNL76/7 feeder cells at a low cell density, and approximately 0.3% cells formed undifferentiated colonies (Figure 1A). The remaining cells did not form colonies. The colonies that formed could be subcloned and expanded in EPSCM to establish stable cell lines. The converted H1 cells (H1-EPSCM) were dissociated to single cells for passaging in the presence of a selective ROCK inhibitor and could be expanded for more than 30 passages on SNL76/7 feeders. H1-EPSCM cells proliferated faster than H1-FGF cells and had a shorter cell cycle time with more cells in S phase and fewer cells in the G0-G1 phase (Figure 1B). It is worth noting that we also attempted to convert the H9 hESC line but without success (data not shown), suggesting heterogeneity in convertibility of hESC lines. These results are not unexpected because of the reported heterogeneity of human PSC lines and their apparent state-specific conversion potential 42, 43, 44, 45. However, we could use PSC reprogramming to generate EPSCM-PSCs; we established stable EPSCM lines from both episomal-based six-factor reprogramming [30] of human neural stem cells and PB transposon-based six-factor reprogramming of dermal fibroblast cells (Figure 1C-D).

Consistent with maintenance of pluripotency, all EPSCM-cultured lines derived from episome-reprogrammed clones (EC), *piggyBac*-reprogrammed clones PB, or H1-converted ESCs (H1), expressed pluripotency genes (*OCT4, SOX2, NANOG, STELLA, PRDM14,* and *FGF4*) at similar levels to H1-FGF PSCs (Figure 1E). However, EPSCM PSCs displayed lower expression of lineage markers (*SOX1, PAX6, T, GSC, GATA6,* and SOX17) (Figure 1F). Immunostaining further confirmed that EPSCM cultures expressed OCT4, SOX2, NANOG, SSEA3, SSEA4, and TRA-1-81 (Figure 1G). Additionally, spectral karyotyping demonstrated genetic stability of the converted and reprogrammed EPSCM lines (Figure 1H). We also functionally confirmed pluripotent differentiation potential of human EPSCM cultures using in vivo teratoma formation assays (Supplementary Figure E2A, online only, available at www.exphem.org).

In human cultures, each inhibitor in the EPSCM was necessary for the maintenance of the undifferentiated pluripotent state. Quantitative gene expression after four passages minus each inhibitor indicated that each component was essential to maintain the expression of pluripotency related genes *(OCT4, NANOG, SOX2)* and/or to prevent expression of differentiation-related genes *(SOX1, PAX6, GSC, T, GATA6, SOX17, CDX2)* (Supplementary Figure E2B, online only, available at www.exphem.org). As in mouse EPSCs [27], the magnitude of effect was greatest for inhibitors of WNT and Src signaling (Supplementary Figure E2B, online only, available at www.exphem.org). We also noted that CHIR99021 was important for the colony forming potential of PSCs in EPSCM after single cell dissociation (Supplementary Figure E2C, online only, available at www.exphem.org).

**Figure 2:**
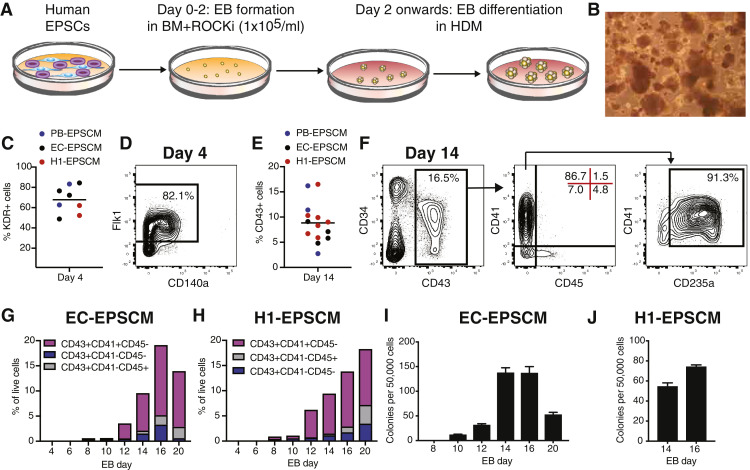
EPSCM PSC differentiation models human hematopoiesis in vitro. (**A**) Schematic of EPSCM PSC differentiation protocol. Human PSCs cultured in EPSCM were dissociated into a single cell suspension for EB formation in basal media (BM) consisting of DMEM/F-12 plus 20% KSR supplemented with ROCK inhibitor (ROCKi) at 1 × 10^5^ cell/mL. After 48 hours, EBs were collected and transferred into human differentiation media (HDM) consisting of Knockout-DMEM plus 20% FBS, transferrin, ascorbic acid, and 2-mercaptoethanol. (**B**) Example image of EBs generated from EPSCM-maintained PSCs. (**C**) Percentage of KDR^+^ cells within day 4 EBs generated from PB-EPSCM, EC-EPSCM, and H1-EPSCM lines using the differentiation strategy described in **A.** Each dot represents an individual differentiation (*n* = 8). (**D**) Example flow cytometry plot displaying expression of KDR and CD140a in day 4 EBs, for differentiations described in **B.** (**E**) Percentage of CD43^+^ cells within day 14 EBs generated from PB-EPSCM, EC-EPSCM, and H1-EPSCM lines using the differentiation strategy described in **A.** Each dot represents an individual differentiation (*n* = 14). (**F**) Example flow cytometry plot displaying expression of CD43 and CD34 (left), CD41 and CD45 (middle), and CD41 and CD235a (right) in day 14 EBs, for differentiations described in **E.** (**G**) Percentage of CD43^+^ subsets between EB day 4 and 20, derived from EC-EPSCM cells using the method described in **A.** Representative of two biological replicates. (**H**) Percentage of CD43^+^ subsets between EB day 4 and 20, derived from H1-EPSCM cells using the method described in **A.** Representative of two biological replicates. (**I**) Average number of CFUs from 50,000 EC-EPSCM-derived EB cells between day 8 and 20. Average of triplicates ± standard deviation. Representative of two biological replicates. (**J**) Average number of CFUs from 50,000 H1-EPSCM–derived EB cells at day 14 and 16. Average of triplicates ± standard deviation. Representative of two biological replicates.

We next assessed whether EB differentiation protocols used for in vitro hematopoietic differentiation of mouse PSCs could be simply applied to human EPSCM-cultured PSCs. As mouse PSCs differentiate via EB formation from a single cell suspension, we initially tested EB formation by human EPSCM-cultured PSCs after single cell dissociation. Additionally, because mouse PSCs undergo in vitro hematopoiesis without addition of recombinant cytokines, we opted to test in vitro hematopoiesis in a simple media composed of KO-DMEM supplemented with FBS, transferrin, ascorbic acid, and 2-mercaptoethanol. However, few EBs formed when we directly transferred PSCs from EPSCM into differentiation media (data not shown). We therefore opted to initially generate EBs in the basal EPSCM (DMEM/F-12 + 20% KSR) supplemented with ROCK inhibitor for 48 hours before transfer to FBS-based differentiation media (Figure 2A), with media refreshed every three days to maintain the EBs (Figure 2B). By the EB day 4 timepoint, KDR^–^ and KDR^+^ cell populations could be resolved (Figure 2C-D), indicating EBs contained mesodermal committed cells [3]. At this time point, 49%-84% of cells were KDR^+^ (Figure 2C), a subset of which coexpressed CD140a (Figure 2D).

To initially assess hematopoietic cell formation, we focused on EB day 14, at which time we could detect 5%–17% cells expressing the pan-hematopoietic cell marker CD43 [46] (Figure 2E), with a subset coexpressing the endothelial and stem/progenitor marker CD34 [47]. Within the CD43^+^ population, distinct subsets could be resolved using hematopoietic cell markers CD41 and CD45; a CD41^–^CD45^–^ population, a CD41^+^CD45^–^ population (which coexpressed CD235a), and a CD41^–^CD45^+^ population (Figure 2F). In human developmental hematopoiesis 4, 48 the CD41^+^CD235a^+^ population has been described as a transient definitive wave of hematopoietic erythromyeloid progenitors (EMPs). By contrast, the CD34^+^CD45^+^ compartment has been suggested to represent definitive wave hematopoiesis 46, 47, 49. As CD43 has been suggested to be the first marker of hematopoietic commitment, the CD43^+^CD41^–^CD45^–^ likely represented early-stage committing hematopoietic cells.

To further characterize human hematopoiesis from EPSCM-maintained PSCs, we performed flow cytometry analysis for hematopoietic cell markers up to day 20 (Figure 2G-H). During EB differentiation, CD43^+^ cells could be identified at day 8, although at low frequencies. The first phenotypic hematopoietic cell types identified were CD43^+^CD41^–^CD45^–^ and CD43^+^CD41^+^ cells (Figure 2G-H), with CD43^+^CD45^+^ cells apparent from day 14 (Figure 2F-H). The percentage of CD43^+^ cells continued to rise after day 14, reaching ∼20% by days 16-20 (Figure 2F-H). Additionally, consistent with the maturation of hematopoietic cells within the EB, by day 20 a subset of CD41^+^ cells started to coexpress CD45, and CD43^+^CD45^+^CD11b^+^ cells could also be identified (Supplementary Figure E3, online only, available at www.exphem.org).

We next investigated hematopoietic colony assay potential during EB differentiation (Figure 2I-J). With the EC-EPSCM PSCs, hematopoietic CFUs could be initially identified from day 10, but highest frequencies of CFUs were identified at day 14 and 16, correlating with the percentage of CD43^+^ cells. Similar timing of CFUs was also from the H1-EPSCM PSCs (Figure 2J), although the frequency was approximately half of the EC-EPSCM–derived EBs.

**Figure 3:**
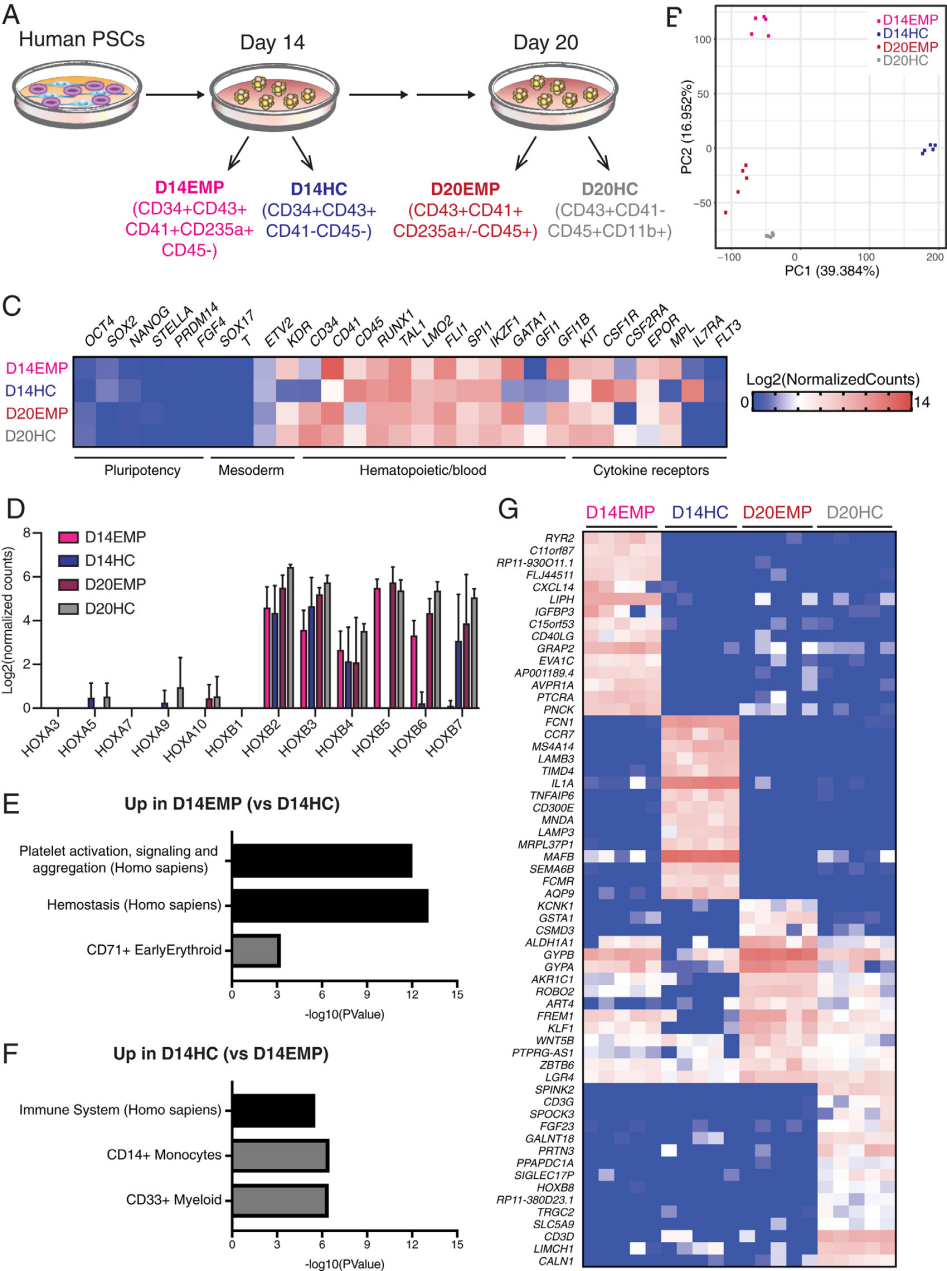
Transcriptional characterization of human EPSCM-derived hematopoietic cells. (**A**) Schematic of human PSC differentiation from EPSCM into EBs and cell populations sorted for RNA-seq analysis from day 14 and day 20 EBs. For each cell population, a total of 50 cells were sorted per RNA-seq sample. Five RNA-seq samples were processed per cell type. (**B**) Principal component analysis of the RNA sequencing of the four cell populations (five replicates per population) described in **A.** (**C**) Heatmap displaying the mean gene expression of various pluripotent and hematopoietic genes (display as Log2[normalized counts] from the RNA-sequencing analysis. (**D**) Log2(normalized counts) RNA-seq gene expression of HOXA and HOXB cluster genes within RNA-seq data sets from **A.** Data are mean ± standard deviation from five samples per cell type. (**E and F**) Bar graphs displaying *p* values from Enrichr analysis [39] for the top 250 differentially expressed genes between D14EMP and D14HC samples within Human Cell Atlas (gray bars) and Reactome 2016 (black bars) annotated gene sets. (**D**) Enrichment for genes upregulated in D14EMP. (**E**) Enrichment for genes upregulated in D14HC. (**G**) Heatmap displaying gene expression of the 15 most uniquely upregulated genes for each cell type within each sample. Gene expression displayed as display as Log2(normalized counts), as in **C.**

Having validated a simple protocol to study human hematopoiesis from EPSCM-maintained PSCs, we next characterized the hematopoietic cell types generated at the transcriptional level by performing RNA-seq (Figure 3A). To better understand the differences between the phenotypically distinct CD43^+^ hematopoietic progenitor cell subpopulations at day 14, we performed RNA-seq (50 cells/sample) on the putative EMPs CD43^+^CD34^+^CD41^+^CD235a^+^ cells (termed D14EMP) and putative definitive-wave CD43^+^CD34^+^CD41^–^CD45^–^ cells (termed D14HC) (Supplementary Figure E4, online only, available at www.exphem.org). Additionally, to further assess their respective lineage differentiation trajectory, we also performed RNA-seq on cells from day 20 EBs, the CD43^+^CD41^+^CD45^+^ cells (termed D20EMP) and CD43^+^CD41^–^CD45^+^CD11b^+^ cells (termed D20HC) (Supplementary Figure E4, online only, available at www.exphem.org).

To initially assess the relationship between these cell samples, we performed principal component analysis (Figure 3B). Sample replicates formed distinct clusters and separated from each other by cell type, indicating that these populations were molecularly distinct. Importantly, the pluripotency TFs that were highly expressed in EPSCM (Figure 1E) were essentially undetectable in all samples (Figure 3C). Instead, all cell types highly expressed hematopoietic markers and TFs [40], although lineage-specific genes, such as *GATA1, GFI1*, and *MPL*, were differentially expressed between cell types (Figure 3C). Although we sorted on surface protein level expression of CD34, the D14EMP and D14HC populations expressed lower levels of *CD34* at the messenger RNA (mRNA) levels. These results indicate that these populations may be in the process of differentiation (and associated downregulation of *CD34* gene expression). By contrast, *CD45* mRNA expression could be identified in D14EMP (which were negative for CD45 based on surface protein expression), consistent with their differentiation trajectory into mature CD45^+^ hematopoietic cells. Of the samples collected, only D20HC expressed *IL7R* and none expressed *FLT3* (Figure 3C), suggesting these phenotypic populations lacked lymphoid potential. Together, these data confirm the hematopoietic commitment during this simple PSC differentiation and are consistent with the notion that these populations represent distinct cell types.

*HOX* gene expression has been recently reported to distinguish yolk sac–like developmental hematopoiesis from aorta-gonad-mesonephros–like developmental hematopoiesis 4, 50, 51. Similar to other in vitro hematopoietic protocols 4, 50, all our cell populations expressed several *HOXB* cluster genes but lacked expression of the *HOXA* gene cluster (Figure 3D). These results suggest that our EB hematopoietic differentiation likely mimics yolk sac–like hematopoiesis. We also evaluated expression of a gene set previously identified by Ng et al. [50] as enriched within in vitro PSC-derived hematopoietic cells. We found that the majority, although not all, of these genes were also expressed within our cell populations (Supplementary Figure E4B, online only, available at www.exphem.org). These results suggest that the hematopoietic cell types generated by our approach are not so dissimilar to those generated by other PSC differentiation protocols.

To further characterize the cell identity of the sorted cell types, we performed differential gene expression analysis (using a stringent cutoff of FDR at 0.05 and a log_2_ fold change of >1). This identified 3,429 differentially expressed genes between the EMP and definitive day 14 populations, whereas only 230 differentially expressed genes between the EMP and definitive day 20 populations (Supplementary Tables E4 and E5, online only, available at www.exphem.org). These results highlight the distinct transcriptional programs operating within different hematopoietic cell types. Gene ontology enrichment analysis (using the top 250 differentially upregulated genes at day 14) identified enrichment of platelet and erythrocyte lineage-associated gene sets in the D14EMP cells (Figure 3E) and enrichment of myeloid/immune-response gene sets in the D14HC cells (Figure 3F). Finally, we searched for unique gene markers within each cell type. This identified erythroid-related genes highly expressed in D20EMP such as *GYPA* and *GYPB,* although they were also found at lower levels in D14EMP and D20HC samples (Figure 3G). Interestingly, lymphoid-related genes were also identified in D20HC (e.g., *CD3G, CD3D*). These results suggest that, as seen during in vivo development [52], CD11b expression may not be restricted to the myeloid lineage. Taken together, these results validate the hematopoietic identity of EPSCM-derived cells.

**Figure 4:**
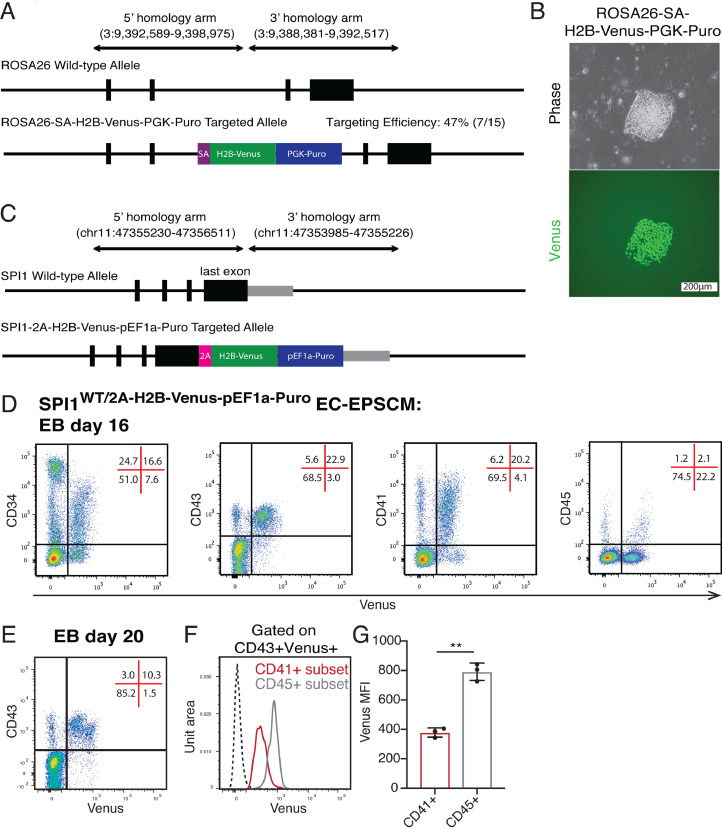
Gene editing in EPSCM affords development of reporter lines. (**A**) Schematic of the human *ROSA26* gene locus and knock-in strategy to insert a Splice Acceptor–H2B–Venus–PGK–Puro cassette by homologous recombination. (**B**) Representative image of Venus expression from a correctly targeted EPSCM PSC line. Of the 15 clones derived from this gene targeting, 7 were correctly targeted. (**C**) Schematic of the human *SPI1* last exon and knock-in strategy to insert a T2A-H2B-Venus-pEF1a-Puro cassette by CRISPR/Cas9-mediated homologous recombination. (**D**) Representative flow cytometry plots for Venus expression from SPI1-T2A-H2B-Venus EPSCM (clone 8) PSC-derived EBs at day 16. (**E**) Representative flow cytometry plots for Venus expression from SPI1-T2A-H2B-Venus EPSCM (clone 8) PSC-derived EBs at day 20. (**F**) Representative flow cytometry histogram for Venus expression within the CD41^+^ (in red) and CD45^+^ (in gray) within the CD43^+^Venus^+^ population, as in **E**, from SPI1-T2A-H2B-Venus EPSCM (clone 8) PSC-derived EBs at day 20. (**G**) Quantification of fluorescence intensity (MFI) of Venus expression from **F**. Data are mean ± standard deviation from three biological replicates. Statistically significant changes (Student's *t* test) denoted by ** *p* < 0.01.

Finally, to expand the utility of our EPSCM differentiation protocol to study human hematopoiesis, we sought to validate gene targeting strategies for generation of reporter PSC lines. To initially assess the efficiency of homologous recombination in human EPSCM cultures, a splice acceptor–H2B–Venus cassette was targeted to *ROSA26* (Figure 4A; Supplementary Figure E5A, online only, available at www.exphem.org). The single-cell cloning efficiency of human PSCs in EPSCM afforded simple clonal expansion of homogenous clones and fluorescence imaging identified a correct targeting efficiency of 47% (Figure 4B). We also exploited the CRISPR/Cas9 system [53] to generate reporter knock-ins in less-permissive gene loci. To expand the utility of EPSCM for studying hematopoiesis, we created a hematopoietic cell commitment reporter line by knocking in a T2A-H2B-Venus reporter into the stop codon of the *SPI1* gene (Figure 4C; Supplementary Figure E5B, online only, available at www.exphem.org). We have previously found that SPI1 expression marks hematopoietic cell commitment in mouse developmental hematopoiesis [33]. Of the nine colonies expanded from this gene targeting, two clones (clones 4 and 8) had correct knock-in (22%) and displayed Venus expression after EB differentiation (Supplementary Figure E5C–D, online only, available at www.exphem.org). However, Sanger sequencing of the untargeted *SPI1* allele identified a 42 bp deletion at the Cas9/sgRNA target site in clone 4 (Supplementary Figure E5E, online only, available at www.exphem.org). We therefore focused further analysis on clone 8, which retained a wild-type allele.

To validate this SPI1 reporter cell line, we determined Venus expression in the various cell populations generated at EB day 16. Consistent with the hematopoietic-restricted expression pattern of SPI1, Venus expression was identified in the majority of CD43^+^ cells, including CD41 and CD45 populations (Figure 4D). Although Venus expression was seen in some CD34^+^ cells, likely hematopoietic committing cells, expression was excluded from the CD34^hi^ CD43^–^ cell population (Figure 4D), consistent with the described endothelial identity of this population [3]. Venus expression was similarly localized to the CD43^+^ population at EB day 20 (Figure 4E). Interestingly, within this population, higher Venus expression could be found in the CD45^+^ subset compared with the CD41^+^ subset (Figure 4F), with an average of twofold more Venus fluorescence (Figure 4G). This differed to the similar mRNA level expression of *SPI1* in our RNA-seq analysis, suggesting post-transcriptional regulation may be regulating cell type–specific SPI1 expression levels [54]. We therefore conclude that gene targeting in EPSCM-cultured PSCs provides an easy strategy to generate reporter lines to study developmental lineage commitment and/or systems to optimize generation of specific cell types.

## Discussion

Here we have reported that EPSCM developed for mouse EPSC culture can also be used to stably maintain human PSCs. Moreover, we have found that human PSCs maintained in EPSCM differentiate into hematopoietic cells using a simple EB differentiation approach. These results mimicked our findings with mouse EPSCs (Supplementary Figure E1, online only, available at www.exphem.org). Epigenomic analysis identified significantly more bivalent domains within mouse EPSCs (vs. 2iLIF PSCs), including several hematopoietic-related TFs. Future studies are needed to assess whether similar changes are found during the conversion of human FGF-PSCs into EPSCM-PSCs.

A key difference between the human EPSCM-PSC EB differentiation protocol described here and standard human FGF-PSC EB differentiation protocols is that this EB formation protocol initiates from a single cell suspension of PSCs, rather than PSC clumps (or reaggregations) 31, 47, 55. However, similar to traditional human PSC in vitro differentiation protocols 31, 47, our new differentiation system appears to mimic developmental yolk sac hematopoiesis. We envisage that the cytokine-free differentiation platform described here will provide the basis to optimize the generation and expansion of PSC-derived blood and immune cell types.

To expand the utility of EPSCM, we have also found that similar to mouse [27], human PSCs cultured in EPSCM also undergo homologous recombination and afford generation of transgenic PSC lines. The efficiency of single cell cloning in EPSCM adds to the ease of generating gene-targeted PSC lines using this approach. We believe this is a major advantage of the protocol described here. As proof of concept for this, we generated a SPI1-2A-Venus reporter line as a tool to study human hematopoiesis. A future application of this reporter line will be the screening for factors that alter the frequency of Venus^+^ cells. Together, these tools provide a useful toolkit to interrogate the biological networks regulating mammalian cell identity and fate decisions [56].

## Conclusions

We have validated EPSCM for the maintenance of human ESCs and iPSCs and found that EPSCM affords spontaneous in vitro developmental hematopoiesis in simple differentiation conditions as well as gene targeting to generate reporter PSC lines. We hope that this platform provides a useful toolkit to study human developmental hematopoiesis. This study also suggests that differentiation analysis of other recently described human PSC culture conditions 19, 20, 21, 22, 23 is warranted. Our results suggest that there may be practical advantages of using these new human PSC cultures over traditional human PSC cultures, both for studying developmental hematopoiesis and for in vitro blood cell production.

## Appendix B Supplementary materials

Image, application 1

Image, application 2

## Footnotes

Supplementary material associated with this article can be found, in the online version, at doi:10.1016/j.exphem.2019.07.003.
